# Murine models to study human NK cells in human solid tumors

**DOI:** 10.3389/fimmu.2023.1209237

**Published:** 2023-06-14

**Authors:** Monica Parodi, Simonetta Astigiano, Paolo Carrega, Gabriella Pietra, Chiara Vitale, Laura Damele, Melania Grottoli, Maria de la Luz Guevara Lopez, Riccardo Ferracini, Giulia Bertolini, Ilaria Roato, Massimo Vitale, Paola Orecchia

**Affiliations:** ^1^Unità Operativa UO Patologia e Immunologia Sperimentale, IRCCS Ospedale Policlinico San Martino, Genova, Italy; ^2^Animal Facility, IRCCS Ospedale Policlinico San Martino Genova, Genova, Italy; ^3^Laboratory of Immunology and Biotherapy, Department of Human Pathology, University of Messina, Messina, Italy; ^4^Dipartimento di Medicina Sperimentale, Università di Genova, Genova, Italy; ^5^Department of Surgical Sciences, Bone and Dental Bioengineering Laboratory, C.I.R Dental School, University of Turin, Turin, Italy; ^6^Department of Surgical Sciences (DISC), University of Genoa, Genoa, Italy; ^7^“Epigenomics and Biomarkers of Solid Tumors”, Fondazione IRCCS Istituto Nazionale dei Tumori, Milan, Italy

**Keywords:** natural killer cells, humanized mice, solid tumor, human-in-mouse model, tumor immunity

## Abstract

Since the first studies, the mouse models have provided crucial support for the most important discoveries on NK cells, on their development, function, and circulation within normal and tumor tissues. Murine tumor models were initially set to study murine NK cells, then, ever more sophisticated human-in-mice models have been developed to investigate the behavior of human NK cells and minimize the interferences from the murine environment. This review presents an overview of the models that have been used along time to study NK cells, focusing on the most popular NOG and NSG models, which work as recipients for the preparation of human-in-mice tumor models, the study of transferred human NK cells, and the evaluation of various enhancers of human NK cell function, including cytokines and chimeric molecules. Finally, an overview of the next generation humanized mice is also provided along with a discussion on how traditional and innovative *in-vivo* and *in-vitro* approaches could be integrated to optimize effective pre-clinical studies.

## Introduction

Natural Killer (NK) cells are Innate Lymphoid Cells (ILCs) playing a crucial role in the anti-tumor and antiviral immunity (1–4). They carry out their function both by influencing and supporting the activity of different immune cell types (including myeloid cells and T lymphocytes), and by directly killing the target cells (5–8). Through the release of chemoattractant factors (CCL3, CCL4, CCL5, HMGB1) and cytokines (IFN-γ, TNFα, and GM-CSF), they can favor recruitment of immune cells, including NK cells (9, 10), and promote Th1-type inflammatory responses, while through the expression of cell death receptors and, especially, the release of cytotoxic granules they exert a potent lytic activity against tumor and virally infected cells. The activation of the different NK cell functions is orchestrated by the cytokine milieu and, more specifically, it depends on the type of cellular interactions involving NK cells. The cell-to-cell contact with normal cells, certain immune cells, or altered cells can give rise to inhibitory, regulatory, or lytic immune synapses respectively (11), with different patterns of engaged NK receptors, including: inhibitory, activating, or cytokine receptors. In different species, the discrimination between normal cells and altered tumor or virally infected cells is carried out through the action of MHC-I-specific inhibitory receptors, and activating receptors recognizing stress-induced molecules or ectopically expressed antigens. In humans, major inhibitory receptors are represented by certain Killer Ig-like Receptors (KIRs) and the CD94:NKG2A heterodimer, while the most known tumor-recognizing receptors include the Natural Cytotoxicity Receptors (NCRs) (NKp46, NKp30, and NKp44), NKG2D, and DNAM-1 (12, 13). Remarkably, the coordinated engagement of some inhibitory and activating receptors, together with cytokine receptors are involved in complex regulatory cross-talks with immune cells.

This picture of NK cell function and mechanics is the result of the many studies that have been carried out *in vitro* over the 90s and across the millennium to dissect single molecular pathways and cellular interactions. These results were then impressively expanded with the advent of new global and in-depth cell analytical approaches, such as the “omics” and “single cell RNA-seq”. Application of these techniques led to the identification and fine molecular characterization of new functional NK cell subsets, as naïve or adaptive NK cells, or tumor-associated NK cells (14–16). Despite such important technical advances have significantly extended the power of human *in vitro* and *ex-vivo* studies, the still open issues on NK cell differentiation, circulation, homing, tissue residency, and tumor penetration cannot disregard the option of the animal studies.

Along the timeline of the NK cell studies, the utilization of murine models proved crucial for the initial evaluation of the real anti-tumor effects of NK cells and gave the first molecular hints on the role of MHC-I recognition (17–20). Additional studies provided supportive information on how NK cells differentiate, acquire and regulate their cytolytic potential (through the “licensing/arming” process), and, even more strikingly, persist *in vivo* as memory-like cells (21–24). These new insights are providing important hints for the selection and preparation of optimized NK cell effectors for anti-tumor immuno-therapies, with promising results in the field of hematological malignancies. On the other hand, the successful exploitation of NK cells in the cure of solid tumors is still limited by the active role of the local tumor microenvironment (TME), which hinders infiltration and effector capabilities of NK cells (25, 26). Several *in vitro* studies have contributed to the fine dissection of the different molecular mechanisms underlying the suppressive/escape properties of the TME. Nevertheless, the murine models are providing the crucial answers on how each of these mechanisms, and its targeting, could impact on the real anti-tumor efficacy of NK cells within the complexity of the TME. Given the importance of this issue, many efforts are being spent to generate ever more effective models to study solid tumors, moving from the full murine to the human-in-mice or humanized models, trying to overcome the not-always obvious problems related to the inter-species differences.

## Mouse models for the study of mouse cancer

Murine models are well-characterized systems useful to gain insights into human biology and various pathological conditions, including cancer. Indeed, through the development of specific strains with stable and well-known genetic background, the murine system offers defined and reproducible experimental conditions without losing the complexity of living organisms.

The initial studies to identify and molecularly characterize the anti-tumor functions of NK cells *in vivo* were performed on mice injected with murine malignant cells. By the use of a C57BL strain and its “beige” variant, characterized by reduced NK cell activity related to the beige-J spontaneous mutation *Lystbg-J*, it was demonstrated the active role of NK cells in contrasting tumor growth (18, 19). Then, experiments based on the injection of H-2 mismatched or H-2 negative tumor cells in mice bearing given H-2 haplotypes provided important information on the role of the MHC-I-specific NK receptors (27, 28). Finally, the development of induced or spontaneous tumorigenesis mouse models combined with the generation of specific gene silencing (KO mice) were crucial to demonstrate the key role of certain activating NK receptors *in vivo*. Thus, for instance, in the model of transgenic adenocarcinoma of the mouse prostate (TRAMP) the targeting of the *klrk1* gene (TRAMP-NKG2D-KO mice) resulted in higher tumor incidence and the development of larger tumors (29). Similarly, the inactivation of the *cd226* gene (DNAM-1-KO mice) favoured the growth of the (3-MethylColanthrene (MCA)-induced fibrosarcoma in BALB/c mice (30). Finally, the inactivation of the *Ncr1* gene affected the ability of C57BL/6 mice to control melanoma and lung carcinoma metastases, or lymphoma induced by tumor cell line injection (31, 32).

The “mouse in mouse” studies provided important hints for the characterization of the role of human NK cells in the context of the solid tumors, as many murine NK cell receptors presented human homologues, and homologies between human and mice also emerged studying the development of NK cells, the regulation of their functional properties, and the acquisition of memory-like features. However, although these models have been exceedingly important in advancing our knowledge, there are limits and functional differences that have to be accounted for, especially, considering the translation of the obtained data to the design of new immunotherapy strategies (33). In this context, it has become ever more important the development of models enabling the study of human environments in the mouse.

## Mouse models for the study of human cancer

The growth of human tissues and cells in a different species (xenotransplant) requires evasion from the immune system to prevent rejection. Xenotransplants became possible after the discovery, in 1962, of the nu/nu spontaneous mutation (34). Nu/nu Nude mice lacked the thymus, showed T-cell deficiency and impaired B-cell functionality, but retained macrophages and NK cells. Another spontaneous mutation affecting the immune system was discovered in 1983 in C.B-17 mice. Such autosomal recessive mutation, named *Prkdcscid*, affects the gene encoding for the protein kinase, DNA activated, catalytic polypeptide (PRKDC) and gives rise to severe combined immunodeficiency (SCID) in the mice. SCID mice (i.e. homozygous *Prkd^scid^* mutants) lacked both T and B cells and had reduced NK cells (35), however their phenotype was “leaky” and clones of functioning B and T cells could randomly develop in young animals (36). Altered immunity was also observed in nonobese diabetic (NOD) mice (37, 38). These mice were obtained by a combination of inbreeding and selective breeding from a progeny of an outbred Jcl:ICR mouse, and were initially selected as a tool to study autoimmune diabetes, given their genetic predisposition to the development of the disease. However, NOD mice were also characterized by defects in the innate immunity, including the NK cell compartment, and were then considered for the preparation of new immunocompromised models. In particular, by crossing C.B-17 *Prkdc^scid^* males with NOD/ShiLtSz females, it was generated the NOD-SCID mouse strain. Transfer of the SCID mutation onto a non-obese diabetic (NOD) background eliminated the leaky phenotype of the SCID model and generated the NOD-SCID mouse strain that rapidly became the best choice for transplant studies using freshly isolated human tumour cells, selected cancer stem cells, or tumour fragments (39, 40). NOD-SCID mice lack T and B lymphocytes and display reduced NK cell function, resulting in increased xenotransplant engraftment. However, they have a limited lifespan (7-8 months), due to the frequent spontaneous development of thymic lymphomas (41). Despite their limits, nu/nu, SCID and NOD-SCID mice have represented the most used strains for xenotransplant of human tumour cell lines in the last 50 years. A great improvement in the rate of human cell engraftment was then obtained with the backcrossing of NOD-SCID mice with either truncated or deleted interleukin-2 receptor common gamma chain giving origin to the NOG (42) or NSG (43, 44) strains, respectively. Specifically, in the early 2000s the NOD.Cg-Prkdc^scid^Il2rg^tm1Sug^ (NOG) strain was developed at the Central Institute for Experimental Animals, Japan, by backcrossing NOD/ShiJic-Prkdc^scid^ mice with IL2rg^tm1Sug^/ShiJic mice, carrying a truncation of the intracellular signalling domain of the IL2 receptor gamma chain. The NOD-SCID gamma NOD.Cg-Prkdc^scid^Il2rg^tm1Wjl^/Szj (NSG) strain, instead, a brand from the Jackson Laboratories (USA), was developed by backcrossing NOD-SCID mice with B6.129S4-IL2rg^tmWjl/J^ (IL2Rγ^null^) carrying the knock-out of the IL2 receptor gamma chain. NSG and NOG mice lack mature T and B cells, NK cells, and complement, have defective macrophages and dendritic cells (DCs) and gave a great impulse to the development of humanized mice models. In particular, these mice represent elective recipients for the functional studies on circulating tumor cells.

Cancer tissue represents a quite complex biological system, with its peculiar microenvironment and heterogeneity of the malignant and non-malignant components (45, 46). Therefore, the strategies to generate human tumor tissues in immunocompromised mice were generally conceived with the aim of reproducing at best such original complexity. A valid approach in this field has been the setting up of Patient Derived Tumour Xenografts (PDX), i.e. the engraftment of small pieces of freshly collected tumour samples in immunocompromised mice. PDXs warrant the conservation of the original tumor components (47–49) and, for that reason, are widely used for maintenance and therapy response experiments. In one study on lung cancer PDX, it has also been shown that lymphocytes present in the transplanted tumor (tumor infiltrating lymphocytes - TIL) could partly reconstitute the immune system in the mouse. Remarkably, in these mice, the combined treatment with IL-15 and PD-1 inhibition could induce tumor regression, which was dependent on human (tumor-derived) NK and T cells (50). Therefore, the model could provide important information on the interaction of the immune system with the tumor in the autologous setting. The use of this model, however, appear to be limited by the possible variability of the TIL component in different engrafted tumor fragments. Moreover, it should be considered that murine stroma can replace human one after two passages in the mice, and a cross-talk between the human tumor component and murine tumor-associated cells can easily take place in these models.

Another approach comes from the observation that certain human tumor cell populations, especially those containing a cancer stem cell component, can generate malignant lesions, partly recapitulating the original tumor complexity, once injected into immunocompromised mice. Following this observation, several human-in-mice tumor models have been reproduced and studied through the injection of selected tumor cells in NOD-SCID or NSG mice. An interesting example is represented by the attempt to reproduce a bone metastasis of human breast cancer or NSCLC in a human bone fragment that has been subcutaneously implanted in SCID mice (51, 52). In the case of NSCLC, it has been possible to identify a subset of human NSCLC cells endowed with metastasis-initiating-cell properties. Those cells, which were marked by the CD133^+^CXCR4^+^EpCAM^-^ phenotype, were shown to colonize the bone implant and to generate tumor lesions.

The immunocompromised mice are optimal recipients for the generation and development of human tumours, but their altered, non-human, immune system represents an issue. Indeed, cancer is a dynamic disease, showing plasticity in its cellular and extracellular components, and its dynamics is often influenced by the interaction with the host and, signally, by the selective pressure of the immune system (46). Therefore, the lack of the human immune system in these murine models represents a limit, both for the attainment/maintenance of reliable tumor units, and for the studies focused on the development of immunotherapy strategies.

For this reason, there has been a flourishing interest in the “*humanized mice models*”: immunocompromised mice transplanted with hematopoietic precursors and/or lymphoid tissues capable of reproducing in the mice several types of human immune cells.

## Humanized mice

Engraftment of human lymphoid cells in SCID mice was first reported in 1988 by Mosier and co-workers, who injected intraperitoneally human peripheral blood mononuclear cells (PBMC) from EBV-positive donors and obtained “*stable long-term reconstitution of a functional human immune system*” (53). However, only human T and B cells could be satisfactorily maintained in these mice, while engraftment of other hematopoietic lineages was not effective. An additional problem was represented by the development in the animals of xenogeneic graft-versus-host-disease (GvHD), which usually took place 4-6 weeks after transfer of huPBMC, thus shortening the available observation time (54, 55). Nevertheless, given its relatively easy preparation, this model has been used over time, and, recently, the generation of NSG mice carrying a deletion of the MHC-I or II genes has been proposed to overcome the problem of xenogeneic GvHD (56).

Encouraging results were also obtained by transferring CD34^+^ human hematopoietic stem cells (HSC) from cord blood into new-born BALB/c Rag2^-/-^γc^-/-^ mice by intrahepatic injection. However, the reconstitution level remained low (57). The use of NOD-SCID mice carrying the deletion of the *Rag-1* or *Rag-2* gene did not change significantly the outcome (58). In these models, T cell function was limited, probably due the absence of human lymphoid organs supporting T cell maturation, selection, and activation. To overcome this problem, in a new study, CD34^+^ HSC were transferred into NOD*-*SCID mice that were previously transplanted with human foetal liver and thymic tissue in the renal capsule (hu-bone marrow-liver-thymus - BLT) (59, 60). hu-BLT mouse model exhibits a full reconstitution of human immune cell repertoire, with T cells capable of mounting HLA-II- and HLA-I-restricted specific responses. These models were initially prepared to assess the immune response to viral infections (namely HIV), and were then adapted for studies of onco-immunology (61). Their employment for the study of NK cells in the tumor, however, appeared to be limited by the fact that BLT-mice developed relative low numbers of, poorly functional, NK cells.

The advent of the next generation murine models is now providing specific tools to study restricted human immune cell types in selected human tumors. These models are generated by using “knock in” and “knock out” strategies in immunocompromised mouse background, and can reach considerable complexity (62). **Table 1** summarizes the main models that can be used for the study of human NK cells *in vivo*. An important issue that is generally considered in the setting of humanized mice regards murine cytokines, which may be poorly expressed in immunocompromised mice and, due to the evolutionary divergence, quite different from their human counterparts. Therefore, several models have been developed expressing human cytokines, generally in NOG or NSG background, with the aim to improve development or persistence of given human immune cells, including NK cells (**Table 1**).

**Table 1:** Summary of “first” and “next” generation immunodeficient mouse strains to study human NK cells in human solid tumors.

Mouse strain	Full name	Features of mouse strain	References when first described
**NOD-SCID**	NOD.C.B-17*-Prkdc^scid^*	- Impaired T and B cell lymphocyte development - deficient NK cell function	(39)
**Hu-BLT**	NOD SCID Rag2^-/-^γc^-/-^	- Co-transplantation of human fetal thymus and fetal liver into the renal capsule of mice - Injection of HSC in mice - T, B, Myeloid development	(59, 60)
**NOG**	NOD.Cg-Prkdc^scid^Il2rg^tm1Sug^	- Truncated intracytoplasmic domain of IL2 receptor gamma chain - Defective mouse NK cell development	(42)
**NSG**	NOD.Cg-Prkdc^scid^Il2rg^tm1Wjl^/Szj	- IL2 receptor gamma chain deficiency - lack mature T cells, B cells and functional NK cells - deficient in cytokine signaling	(43, 44)
**NOG-IL2**	NOD SCID Il2rg^null^ hIL2 tg	- Similar to NOG - Transgenic expression of human IL2 - human NK cell proliferation	(63)
**NOG-IL15**	NOD SCID Il2rg^null^ hIL15 tg	- Similar to NOG - Transgenic expression of human IL15 - human NK cell proliferation - human NK cells produce granzyme A and perforin upon stimulation	(64)
**NSG-IL15**	NOD SCID Il2rg^_^/^_^ hIL15 tg	- Transgenic expression of human IL15 - human NK cell proliferation and maturation - human NK cells produce granzyme A and perforin	(65)
**NSG-IL7-IL15**	NOD SCID Il2rg^_^/^_^ hIL7 KI hIL15 KI	- Transgenic expression of human IL15 and IL7 - Efficient development of human NK cells - Human NK cells undergo maturation process and exhibit cytotoxicity	(66)
**NSG-SGM3**	NOD SCID Il2rg^_^/^_^ hIL3/hGMCSF tg hSCF tg	- Transgenic expression of human IL3, GM-CSF and SCF - Development of human myeloid cells (compared to NSG) - Increased levels of human NK cells	(67, 68)
**SRG-15**	Balb/c x 129 Rag2^_^/^_^ Il2rg^_^/^_^ hSIRPA KI hIL15 KI	- Expression of human SIRPa enables mouse phagocytes to tolerate human cells - Development of human ILC1 and NK cell subsets	(69)
**MISTRG**	Balb/c x 129 Rag2^_^/^_^ Il2rg^_^/^_^ hMCSF KI hIL3/hGMCSF KI hSIRPA KI hTHPO KI	- Development of functional human myeloid cells and NK cells	(70)
**BRGSF**	Balb/c Rag2^_^/^_^ Il2rg^_^/^_^ SIRPA^NOD^ Flk2 ^_^/^_^	- Development of functional human myeloid cells and NK cells	(71)

NOD, nonobese diabetic; Scid, severe combined immunodeficient; Il2rg, interleukin 2 receptor gammal; prkdc, protein kinase DNA-activated catalytic polypeptide; BLT, Bone Marrow-Liver-Thymus; hIL2 tg, humaninterleukin 2 transgenic; hIL15 tg, human interleukin 15 transgenic; hIL7 KI, human interleukin 7 knock-in; HSC, haematopoietic stem cells; hIL3/hGMCSF, human interleukin 3/human Granulocyte-macrophage colony-stimulating factor knock-in; hMCSF KI, macrophage colony-stimulating factor knock-in.

Katano et al. established transgenic NOG mice sub-strains expressing either human IL-2 (NOG-hIL-2 Tg) or human IL-15 (NOG-hIL-15 Tg) (63, 64). In NOG-hIL-2 Tg mice, human cord blood-derived HSCs could mature and give rise to differentiated KIR^+^ NK cells expressing IL-2–activated phenotype; while in NOG-hIL-15 Tg mice, the high levels of IL-15 in the blood could support survival of NK cells from transferred human PBMC. hIL-15 has also been expressed in NSG, and the strain NSG-IL-15 Tg is now commercially available (65). Recently, to improve the development of human NK cells, Matsuda et al. created hIL-7 and hIL-15 double knock-in (hIL-7xhIL-15 KI) NSG mice (66). Compared to NSG mice, these mice showed increased ability to develop human NK cells after engraftment of human hematopoietic stem cells. A hIL-15 and human signal regulatory protein alpha (hSIRPα) double knock-in mouse on a Rag2^-/-^ il2rg^-/-^ background (SRG-15) was described to support efficient development of innate lymphoid cell subsets and NK cells, including tissue resident cells (69, 72). Even more complex knock in mice have been generated that express different combinations of human factors such as IL-3, IL-15, GM-CSF, M-CSF, SIRPα, and thrombopoietin: NSG-SGM3, MISTRG and BRGSF (see **Table 1**). On the whole, these latter strains support the development of human myeloid cells, which in turn can produce IL-15 and improve development of functional human NK cells (67, 68, 70, 71). Finally, a potentially interesting humanized model is also represented by the Nonirradiated NOD.B6.SCID Il2rg^-/-^ kit^w41/w41^ (NBSGW) mouse, which was obtained by the crossing of NSG strain with the C57BL/6.-Kit^W-41J^/J strain (73). This model has the interesting advantage of supporting the human HSC engraftment without the need for pre-transplant conditioning (i.e. γ-irradiation), which could negatively affect the host microenvironment for the HSC development (74). An ovarian cancer PDX has been now developed in NBSGW mice, engrafted with human cord blood-derived HSC, to study the tumor immune environment within the peritoneal cavity (75). To date, such NBSGW model has not been used yet to study human NK cells.

Some of the above-mentioned next generation humanized mice have been assayed in different xenogeneic solid tumor models to study various immune cell types, especially T cells and myeloid cells, and only rarely NK cells (**Table 2**). Indeed, as yet, the large majority of the studies on NK cells have been done on “traditional” NOG or NSG mice. Nevertheless, the strategy of transferring human NK cells in such immunocompromised mice proved effective to evaluate which NK cell preparation, or way of cell activation, would be more suitable to overcome the tumor escape mechanisms and to deliver efficient NK cells into the tumor niches.

**Table 2:** Next-generation humanized mice used in preclinical studies for solid tumors.

Humanized mouse model	Solid tumor engrafment	Studied human immune cells	References
**NOG-IL2**	Melanoma Uveal and cutaneous melanoma Cutaneous melanoma	TIL CAR T TIL	(76) (77) (78)
NOG-IL15*	-	-	–
**NSG-IL-15**	Human sarcoma Melanoma Non-small-cell-lung-cancer	NK cells T cells, NK cells T cells, NK cells	(79) (65) (80)
NSG-IL7-IL15*	-	-	–
**NSG-SGM3**	Head and neck cancer (HNC) Clear-cell renal cell carcinoma Metastatic breast cancer Metastatic melanoma Melanoma Atypical teratoid/rhabdoid tumors and central nervous system primitive neuroectodermal tumors Glioblastoma Ovarian cancer Ovarian cancer	T cells CAR T cells Myeloid cells Myeloid cells Dendritic cells CTL T cells Macrophage T cells	(81) (82) (83) (84) (85) (86) (87) (88) (89)
SRG-15*	–	–	–
MISTRG*	–	–	–
BRGSF*	–	–	–

*Humanized mouse models not yet employed in preclinical studies.

## Human NK cell transfer in human-in-mice tumor models

As mentioned above, the human-in-mice tumor models, combined with human NK cell transfer, represent a suitable mean to assess the real effects of NK cells at the tumor site. Through these studies, it is possible to gain knowledge on spatial localization of NK cells within the tumor, and understand their impact on stroma, tumor cells and other immune cell populations. Likewise, it is possible to characterize phenotype and function of tumor-infiltrating NK cells, and, also, to gain information on their persistence. Therefore, on the whole, these models offer a platform to test possible approaches to optimize survival, function, and safety of adoptively transferred human NK cells. As described below, the animal model receiving human NK cells is generally represented by the NSG mice.

### Study of NK cells exposed to cytokines

One of the major limitations to the efficacy of adoptive NK cell therapy is represented by the poor tumor-infiltrating capacity of transferred NK cells (90). The generation of sufficient NK cell numbers for adoptive immunotherapy represents an additional issue. Several strategies, often involving the use of priming cytokines, are employed to expand ex vivo NK cells exhibiting distinct phenotypes, which can be correlated with their anti-tumor potency *in vitro* and *in vivo* (91). Cytokines are then used *in vivo* to sustain persistency and activation of transferred NK cells.

IL-2 has been the most commonly used cytokine to expand NK cells ex vivo and to boost proliferation of adoptively transferred NK cells *in vivo* (92–95). These IL-2-based therapies, however, generally demonstrated poor efficacy at the bed side, even showing negative side effects. The limits of IL-2 were essentially related to its ability to induce Tregs (with consequent suppressive effects on NK cells) (96–98) and to promote Activation Induced Cell Death (AICD) on NK cells. This latter aspect could be particularly relevant in the context of the solid tumors, as IL-2-primed NK cells undergo AICD following interaction with endothelial cells, with a possible negative impact on extravasation and tumor infiltration (99). Finally, it cannot be disregarded that IL-2 can contribute to the vascular leak syndrome, an important adverse effect that limits its use in therapy (100). In the attempt to improve suitability of IL-2 for immunotherapy, engineered IL-2 molecules (termed superkines) have been developed. Compared to IL-2, IL-2superkines induced superior expansion of cytotoxic T cells, more prolonged and more intense NK cell activation, and improved anti-tumour responses in different syngeneic mouse models (101–103). These studies, however have not yet been conducted using human-in-mice models.

Combining IL-2 with other cytokines to pre-activate NK cells before transfer could improve NK cell efficiency, limiting the IL-2-related negative effects. A candidate for such approach may be IL-21, which has been shown to induce expansion of non-terminally differentiated CD56^bright^ NK cells (104) and functional NK cell maturation (105, 106). The efficacy of human NK cells that were expanded ex vivo using feeder cells + IL-2 + IL-21 was analysed in NSG mice injected with a human melanoma cell line. Adoptive transfer of such pre-activated NK cells resulted in increased *in vivo* persistence, as compared to NK cells pre-treated with IL-2 alone, and in significant inhibition of melanoma-induced lung metastases (107).

IL-15 has been proposed as valid alternative to IL-2 to stimulate NK cells for therapeutic protocols. IL-15 and IL-2 are structurally similar, share two of the three subunits of their receptors (namely, IL-2Rβ/CD122 and γ_c_/CD132), and are both capable of activating NK cells. However, the presence of distinctive receptor alpha subunits (IL-15Rα/CD125, and IL-2Rα/CD25) accounts for their different behaviour *in vitro* and *in vivo*. Thus, for example, IL-15 supports NK cell survival, proliferation and effector functions, but, different from IL-2, it has no stimulatory effect on the CD25+ Tregs (108). In addition, IL-15 can be associated to the IL-15Rα and trans presented to NK and T cells by different immune and non-immune IL-15Rα+ cells (109, 110). In different pre-clinical models of solid tumors, IL-15, alone or in combination with additional cytokines (i.e. IL-2, IL-12, and IL-18), has been assayed to stimulate human NK cells before transfer, and/or to support human NK cell persistency after transfer. For example, in a study aimed at evaluating the anti-tumor properties of human NK cells generated *ex vivo* from HSPC, recombinant IL-15 was infused together with NK cells in NSG mice bearing ovarian carcinoma, and this infusion was then followed by a further boost of IL-15 (111). The study, showed efficacy of HSPC-derived NK cells in reducing tumor burden, and was then followed by a clinical trial (NCT: 03539406), which is still ongoing. In this study, patients with recurrent ovarian cancer, pre-treated or not with chemotherapy, are infused intra-peritoneally with allogeneic NK cells generated from UCB CD34+ HSPC. Once completed, this trial will give crucial information on feasibility, safety and toxicity of this innovative therapeutic approach. In a xenografted mouse model of colon rectal cancer (CRC), human NK cells were first expanded *in vitro* in the presence of IL-15 and IL-2, then were transferred into the mice combining infusion of NK cells and IL-2. Further boosts of IL-2 were given to the mice to maintain NK cells *in vivo*. NK cell transfer induced a delay in tumor growth at an early stage (112).

To improve biological activity and to extend *in vivo* half-life of IL-15 (≈40 min), a super-agonistic molecular complex has been developed that combines mutated activating IL-15, the trans-presenting IL-15Rα sushi domain and IgG1-Fc (113). Such super-agonist, termed N-803 (also known as ALT-803) has been tested in an ovarian carcinoma model, where it was demonstrated to promote HPC-NK cell expansion and functionality (114, 115). First reported clinical trials (NCT: 01885897) (NCT: 01727076) of ALT-803 in cancer patients revealed that it is well tolerated and stimulates NK cell activation and expansion and CD8+ T cells, but not Tregs (116, 117).

IL-15 is also the main component of the IL-12/15/18 cytokine cocktail known to induce the so-called cytokine-induced memory-like (CIML) NK cells. These cells, which can be easily induced *in vitro* from PB NK cells, display enhanced anti-tumor effector functions that can be preserved *in vitro* and even increased *in vivo* (118–120). CIML-NK cells have been initially considered for the cure of hematologic malignancies, and clinically assessed for the immunotherapy of acute myeloid leukemia (AML) (NCT: 01898793) (118, 120). Interestingly, in this clinical study CIML NK cells were shown to persist in the patients, and further differentiate. With regard to solid tumors, CIML NK cells have not yet been assessed in clinics, however they revealed potent anti-tumor activity after transfer in xenogenic melanoma and ovarian cancer NSG mouse models (121, 122). As CIML NK cells have been shown to express CD25, mice bearing the melanoma xenograft were given serial boosts of IL-2 to support persistency and activation of transferred NK cells.

An additional interesting anti-tumor effector lymphocyte population is represented by Cytokine Induced Killer (CIK) T cells. These effectors, which can be expanded *in vitro* by exposure to IFN-γ and IL-2, are characterized by the expression of the CD56 NK cell marker and the activating NKG2D receptor. Remarkably, NKG2D supports their TCR-independent anti-tumor activity, which has been demonstrated both *in vitro* and in xenogeneic tumor models developed into NOD-SCID mice (123–125). Given the promising results obtained *in vitro* and *in vivo*, the use of CIK could contribute to innovative clinical approaches for both hematologic and solid malignancies (126, 127).

### Study of CAR-NK and NK cell engagers

Innovative tools to enhance and address anti-tumor NK cell function are represented by engineered chimeric activating receptors (CARs), whose expression is induced on NK cells by transduction protocols to generate CAR-NK, or by multivalent soluble molecules, which are developed as NK cell engagers. In both the cases, the effectiveness of the effector molecules or cells has been evaluated in xenogeneic tumor models.

CARs are generally constituted by a single-chain Fragment variable (scFv), recognizing a specific tumor-expressed antigen, combined with an intracytoplasmic tail of activating transducing molecules (such as the CD3ζ chain). Once expressed on transduced T or NK cells, CARs drive recognition of tumor cells and the subsequent triggering of the effector functions. CARs have been initially set to enhance and address T cell anti-tumor activity and several studies have already been done on these engineered effectors. Therapy with CAR-T, however, appears to be still hampered by several issues, including T cell exhaustion, the appearance of suppressive/regulatory responses, the induction of the cytokine release syndrome (CRS), and even GVHD (128). Given their features, NK cells could be advantageously employed to generate CAR-effectors with minimized side effects.

CAR-NK have been developed especially for hematological malignancies (129, 130), while for solid tumors the studies are still limited. An interesting CAR-NK effector targeting solid tumors has been prepared by transducing *ex-vivo* expanded human NK cells to express the DAP-12-anti-HLA-G CAR. HLA-G molecules are ligands for the inhibitory immune receptors, LILRB1 and LILRB2 (also known as ILT2 and ILT4) (131), and are frequently expressed by solid tumors, therefore, anti-HLA-G CAR-NK can both target tumor cells and relieve immunosuppression. These anti-HLA-G CAR-NK showed tumor cytotoxicity in orthotopic xenograft models of triple negative breast cancer and glioblastoma, developed in NSG mice (132).

Several bivalent or trivalent NK cell engagers have been synthesized and defined with different acronyms depending on the used technical platform and the originating lab. Bi- or tri-specific killer engagers (BiKE or TriKE) consist of a single-chain Fragment variable (scFv) targeting tumor specific antigens, a scFv targeting activating receptors on NK cells (generally CD16), and (in the case of TriKEs) an additional domain generally targeting cytokine receptors to support activation and survival of NK cells (133, 134). The therapeutic efficacy of BiKEs/TRiKEs with different tumor antigen specificities has been demonstrated in various xenograft tumor models, using NSG recipients and evaluating transferred human NK cells (135–138). In a study, the authors analysed the effectiveness of both the engager (which was the bi-specific anti-CD30/CD16 antibody) and of different NK cell effectors, by transferring into the mice either CIML-NK or cord blood-derived NK cells (139).

Recently, a trifunctional NK cell engager (NKCE) triggering simultaneously NKp46 and CD16 on NK cells and targeting a tumor antigen has been developed (140). An upgrade of this engager is then represented by the tetraspecific antibody-based natural killer cell engager therapeutics (ANKETs), which adds the ability to engage IL-R2b to the previous multiple specificity (141). For the preclinical studies, engagers with surrogate anti murine NKp46 were generated and evaluated for their capability to trigger murine NK cells in SCID mice injected with the RAJI or other human lymphoma cells. In some cases, the RAGko huNKp46Tg mice were used to better track NK cells in the tissues, as in these mice NK cells expressed both the human and the murine NKp46, and could be stained by anti-human NKp46 abs without the interference of the anti-murine ANKET.

Several engagers proved promising in the preclinical models, so that phase I/II clinical trials in advanced solid tumors are ongoing (133).

### Other ways to enhance NK cell activity to solid tumors

As mentioned above, the use of irradiated feeder cells combined with cytokines represents a possible strategy to obtain large-scale expansion and activation of NK cells. A recently proposed approach involves, as feeder cells, the NK-92 cell line engineered to express OX40L and to secrete neoleukin-2/15, an artificial peptide that binds with high affinity the human IL-2Rβγ complex. These engineered NK-92 cells were irradiated and used to expand ex vivo-derived human NK cells, which were then transferred in three different xenogeneic tumor mouse models: lung, liver, or ovarian cancers, all developed into NOD-SCID mice. In these experiments, NK-92-induced NK cells showed stronger capability to infiltrate the tumors and a higher antitumor effect compared to NK cells expanded with IL-2 (142).

Chemical approach to increase the antitumor activity of NK cells was reported by Choi et al. who showed how 25kDa branched polyethyleneimine (25KbPEI) could enhance cytotoxicity and migration properties of human NK cells. 25KbPEI-induced NK cells were transferred into xenogeneic breast and ovarian cancer models, which were developed in SCID/nude and NSG mice respectively. In both models 25KbPEI-induced NK cells were demonstrated more effective than IL-2-induced NK cells in infiltrating the tumor and limiting its growth (143).

### Study of the strategies to sensitize tumor cells to NK cell activity

More recently, human tumor xenografts in NSG mice have also been used to evaluate strategies directly targeting the tumor to increase its susceptibility to NK cells. Most strategies are focused on the study of genotoxic or non-genotoxic agents acting on the DNA-Damage-Response (DDR) or related pathways, which lead to increased expression of activating NK Receptor ligands (144). These agents are first evaluated *in vitro* and then validated *in vivo*. Thus, for example, in a xenogeneic neuroblastoma model, mice were given nutlin3a, a non-toxic p53-activating molecule that was demonstrated to increase expression of NKG2D- and DNAM-1-ligands on tumor cells. These mice, compared to those receiving vehicle alone, showed increased NK cell infiltration in their tumor xenografts and reduced tumor growth following human NK cell transfer (95). Analogously, in a xenogeneic ovarian carcinoma model, it has been demonstrated that the chemotherapeutic drug gemcitabine increased the expression of NKG2D ligands and death receptors on tumor cells, and the adoptive transfer of NK cells in combination with gemcitabine additively decreased ovarian cancer growth (145). Finally, in a recent study, combined high-dose radiotherapy and adoptive human NK cell transfer resulted in improved tumor control over monotherapies in NSG mice engrafted with melanoma and pancreatic tumor cells. Such improvement, however, appeared to be related to the radiotherapy-induced CXCL8 release by tumor cells and subsequent recall of CD56dim cytotoxic NK cells (146).

## Final considerations and future development

The increasingly sophisticated analytic tools for the *in-vivo* and *ex-vivo* characterization of tumors are improving clinical decision making, and also, provide means for conducting research directly in the patients. These new approaches add support to the preclinical research but do not replace its fundamental phases, which comprise the studies *in vitro* to dissect molecular and cellular processes, and the validation studies *in vivo*.

The “traditional” 2D *in vitro* cultures address important questions on specific biological, genetic and epigenetic features and on the direct cellular effects of drugs or cytokines, while animal models are amply used to evaluate the actual significance of the dissected processes and to test therapeutic efficacy of drugs and dose-limiting toxicity of clinical treatments. On the other hand, the animal studies are generally expensive, time-consuming, and, for years, they have been based on a limited range of models (147, 148). Furthermore, there are ethical issues, which, for the animal welfare, limit the use of animals to what is strictly necessary. The principle of the 3Rs (Replacement, Reduction, and Refinement) is presently applied in all projects involving the use of animals. In particular, the Replacement is supported by the development and use of predictive and robust models and tools, based on the latest *in vitro* technologies, to address important scientific questions (149).

In this context, the setting of the 3D cultures has been proposed as an important tool for the development of innovative *in vitro* assays and models of neoplastic cell growth with potentially high clinical relevance. By the setting of 3D models, it is tempting to partly reproduce the complexity of the TME, in terms of extra-cellular matrix, cell types, vasculature, and oxygen, nutrients and catabolites distribution. If fully realized, these 3D tumor models would be a powerful mean to study a number of tumor-related processes, to validate specific findings, and to test the efficacy/toxicity of drug treatments, replacing the employment of animals (150, 151). This goal, however, is presently far to be achieved, as it is not easy to set a reliable balance among all the different fundamental components within a single *in vitro*-generated structure.

**Figure 1:**
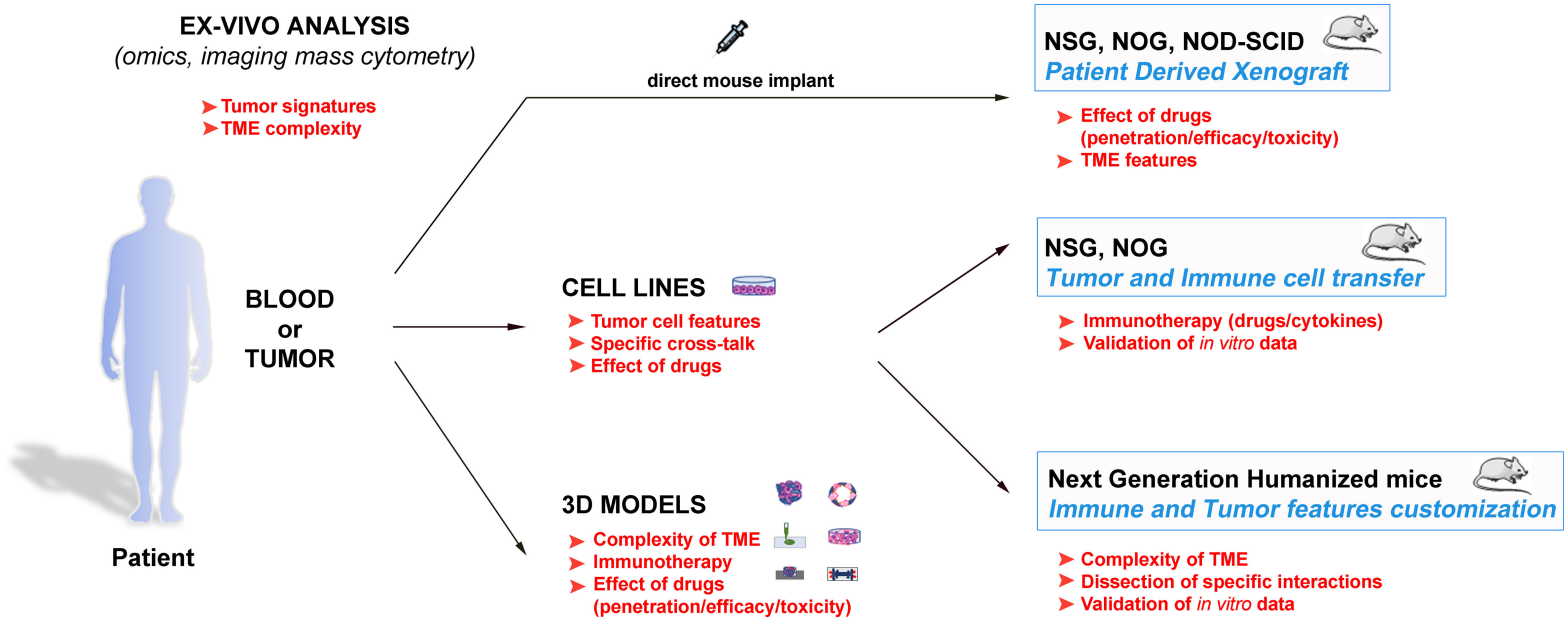
Schematic representation of the different available approaches to get insights in the tumour biology, and to design innovative and personalized therapies. These strategies span from the direct evaluation of the patients’ samples with advanced analytic techniques to the tumour complexity reproduction and the data validation in both *in vitro* and *in vivo* models. The main goals of the various approaches are indicated in red, and suggest how they can be inter-connected to obtain optimal results.

Therefore, the employment of animals remains indispensable for the preclinical studies, especially considering the recent development of the next generation humanized mouse models (65, 76–79, 81–89) (**Table 2**). Indeed, these models can reproduce ever more accurately the complexity of the immune response within the TME, and also offer the interesting perspective of dissecting *in vivo* crucial frames of such complexity. In this context, these models also represent suitable tools to study the behaviour of human NK cells. In conclusion, a powerful strategy to efficiently struggle against cancer should consider the wise integration of the different available tools to gain precise data on the single malignancies, to dissect molecular and cellular pathways, and to evaluate new related therapeutic strategies (**Figure 1**). In this scenario, in which different strategies are connected, the animal models maintain a pivotal role.

## Author contributions

MP, SA, MV, and PO contributed to conception and the outline of the review article. All authors contributed to the article and approved the submitted version.
